# Morphometric Evaluation of the Human Scapula According to Sexes in a Southeastern Brazilian Population

**DOI:** 10.7759/cureus.113163

**Published:** 2026-07-22

**Authors:** Fernando Akio L Miyazaki, Vitoria D Cruz, Camila C Furlan, Alexandre R Freire, Felippe B Prado, Ana Cláudia Rossi, Beatriz C Ferreira-Pileggi

**Affiliations:** 1 Anatomy, Centro Universitário Einstein de Limeira, Limeira, BRA; 2 Biosciences, Universidade Estadual de Campinas (UNICAMP) - Faculdade de Odontologia de Piracicaba (FOP), Piracicaba, BRA; 3 Biosciences/Anatomy, Universidade Estadual de Campinas (UNICAMP) - Faculdade de Odontologia de Piracicaba (FOP), Piracicaba, BRA

**Keywords:** human anatomy, morphology, morphometry, scapula, sexual dimorphism

## Abstract

Human bones exhibit sexual dimorphism, and male and female characteristics are expressed through morphological and metric differences. The aim of the present study was to evaluate the human scapula using morphometric methods, comparing the sexes and antimers in a southeastern Brazilian population. The sample included 88 scapulae, 48 from males and 40 from females. Six measurements were taken on each scapula. The morphometric values were tabulated and statistically analyzed. The analysis of continuous variables comparing the different sexes (male vs. female) and antimers (left and right) with two-way ANOVA followed by Sidak’s multiple comparisons test demonstrated statistically significant differences between sexes for most of the evaluated scapular measurements, with higher values consistently observed in male scapulae. In contrast, no statistically significant differences were identified between the right and left antimers within the same sex. The only parameter that did not show significance between the sexes was the maximum width of the acromion process (MWAP). In conclusion, the findings of the present study indicate that the morphometric measurements of human scapulae from a southeastern Brazilian population demonstrate evident differences between sexes, while maintaining bilateral similarity between antimers within each sex.

## Introduction

The scapula is a bone from the shoulder joint and is involved by several muscles which originate or insert into it [1, 2]. Given the complexity of this bone, it is associated with various shoulder pathologies [3]. Knowledge of the morphology and morphometry of the scapula can help in the identification and treatment of shoulder joint diseases as well as in the development of implants for the joint, in addition to optimizing surgical techniques and improving clinical results [3, 4]. It is known that understanding the morphometry of the patient's shoulder leads to the success of shoulder surgeries, and this can differ between different populations [5, 6].

Human bones exhibit sexual dimorphism, and male and female characteristics are expressed through morphological and metric differences [7, 8]. The fact that the scapula is protected by the various muscles related to it allows it to be found in forensic scenes, representing an alternative to aid in sex determination, which also justifies the importance of studying it [1, 2, 8, 9]. 

Özer et al. [9] state that patterns of sexual dimorphism vary between populations, which requires a specific study for each population individually. Therefore, studies evaluating sexual dimorphism of the scapula have been carried out in several populations, such as a sample of the indigenous population of Guatemala [7], Anatolia [9], the United States of America [10], Iran [11], the Hispanic population [12], India [13], Mexico [1], Japan [14], Chile [2], Greece [8, 15], Turkey [16], Italy [17], Thailand [18] and Portugal [19].

There is also a study performed on a sample of the population of northeastern Brazil [20]. However, it is known that miscegenation in Brazil has been intense, and this means that the Brazilian population does not present well-defined ethnic and racial patterns [20]. There are no studies assessing the morphometric differences of the scapula between the sexes in samples of the population of southeastern Brazil found in the current literature. Given the variability in bone morphology among different populations, even within Brazil, the aim of the present study was to evaluate the human scapula using morphometric methods, comparing the sexes in a southeastern Brazilian population.

## Materials and methods

The study was approved by the Research Ethics Committee of the Centro Universitário Einstein de Limeira (CAAE: 83023624.1.0000.5424).

Sample

The bones evaluated belong to the "Human Bones, Teeth and Cadavers" Biobank of the Faculdade de Odontologia de Piracicaba at the Universidade Estadual de Campinas (FOP/UNICAMP). The study sample included 88 scapulae, 48 from males (24 right and 24 left) and 40 from females (20 right and 20 left). Right and left scapulae were analyzed separately for each sex and were not combined into a single group. The age range was between 20 and 70 years.

The sample size was based on the availability of scapulae within the osteological collection, resulting in a convenience sample. Owing to the limited number of specimens per group, balanced group distribution was not achieved. 

Inclusion and exclusion criteria

The inclusion criteria comprised the use of intact human scapulae with identification and without macroscopic deformities, fractures, or any other pathological or surgical alteration. The exclusion criteria were human scapulae without identification, from individuals with syndromes or with any anatomical abnormalities in the region of interest, as well as individuals with plates and screws or any other metallic artifact near the region.

Data collection

After sample selection, scapular morphometry was performed. All measurements were obtained using a digital caliper (Mitutoyo, model 500-196-20B, 150 mm, Japan) and an osteometric table. The instruments were checked and calibrated according to the manufacturer’s specifications.

**Figure 1:**
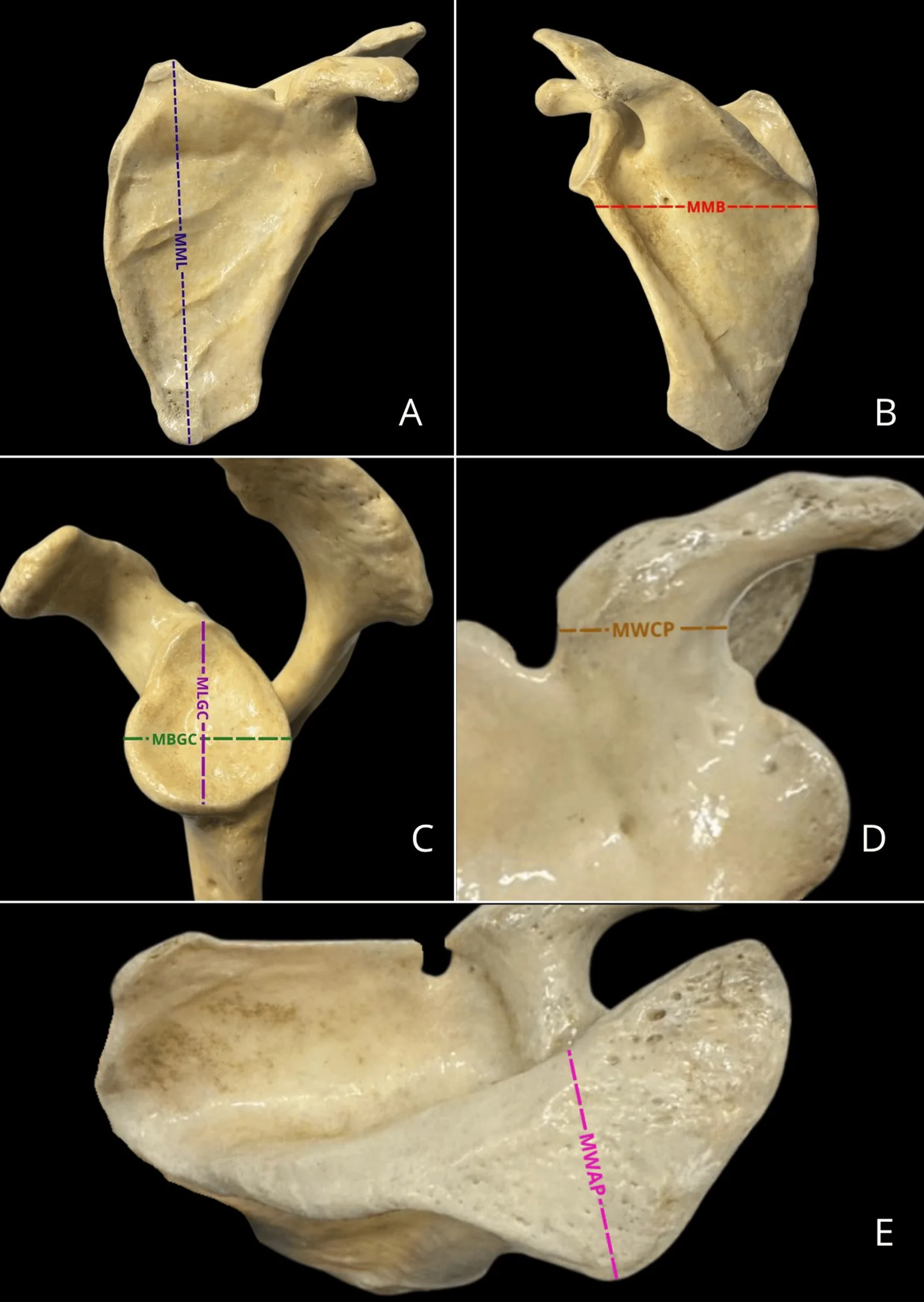
Morphometry of the scapulae. A) Maximum morphological length (MML). B) Maximum morphological breadth (MMB). C) Maximum length of the glenoid cavity (MLGC) and Maximum breadth of the glenoid cavity (MBGC). D) Maximum width of the coracoid process (MWCP). E) Maximum width of the acromion process (MWAP).

Six linear measurements were taken from each scapula, based on previously described methods [17] and illustrated in Figure 1. The following parameters were recorded:

(a) Maximum morphological length (MML), the maximum linear distance between the superior angle and the inferior angle of the scapula (Figure 1A); (b) Maximum morphological breadth (MMB): Maximum linear distance between the medial border and the lateral border of the scapula (Figure 1B); (c) Maximum length of the glenoid cavity (MLGC): Maximum linear distance between the superior and inferior margins of the glenoid cavity (Figure 1C); (d) Maximum breadth of the glenoid cavity (MBGC): Maximum linear distance between the anterior and posterior margins of the glenoid cavity (Figure 1C); (e) Maximum width of the coracoid process (MWCP): Maximum linear distance between the medial and lateral margins of the coracoid process (Figure 1D); (f) Maximum width of the acromion process (MWAP): Maximum linear distance between the anterior and posterior margins of the acromion process (Figure 1E).

Measurements were carried out by two observers. Intra- and interobserver rates were calculated by remeasuring 20 randomly selected scapulae (10 male and 10 female) for each measurement variable.

Statistical Analysis

The intraclass correlation coefficient was calculated to determine the agreement between the measures of the two researchers. The morphometric values were tabulated and statistically analyzed. The normality of the data was assessed using the Shapiro-Wilk test, and the homogeneity of variances was assessed using Levene's test. A two-way analysis of variance (ANOVA) was performed to compare scapular measurements between the right and left antimers, as well as between females and males. The analysis allowed for the evaluation of the main effects of antimer and sex, as well as possible interactions between these variables. When significant effects were identified, multiple comparisons were performed using Šídák's post hoc test.

Statistical analyses were performed using GraphPad Prism v. 10 software (San Diego, CA, USA). The significance level was set at P<0.05.

## Results

The intraclass correlation coefficient for continuous measures was 0.94 (95% CI: 0.75-0.99). Based on the statistical analyses performed, this study identified differences in the measurements assessed between sexes (male and female) and between the right and left scapulae (Table 1). No statistically significant differences were observed between the right and left antimers within the same sex in any of the six measurements. However, significant differences were detected between males and females for some of the evaluated measurements.

**Table 1:** Mean and standard deviation values for each morphometric variable according to sex and side.

Variable (mm)	Female Left	Female Right	Male Left	Male Right
MML	136.50 ± 9.80	135.60 ± 7.98	153.29 ± 11.59	153.75 ± 10.03
MMB	89.06 ± 7.92	88.30 ± 6.89	98.31 ± 9.52	99.52 ± 7.86
MLGC	35.00 ± 1.96	35.18 ± 2.87	38.95 ± 2.92	39.70 ± 2.80
MBGC	24.15 ± 1.87	25.46 ± 2.30	28.29 ± 2.49	29.67 ± 2.53
MWCP	22.20 ± 3.30	21.73 ± 2.03	23.99 ± 3.47	24.25 ± 4.52
MWAP	26.68 ± 5.56	27.94 ± 5.06	29.03 ± 4.84	30.10 ± 4.90

**Figure 2:**
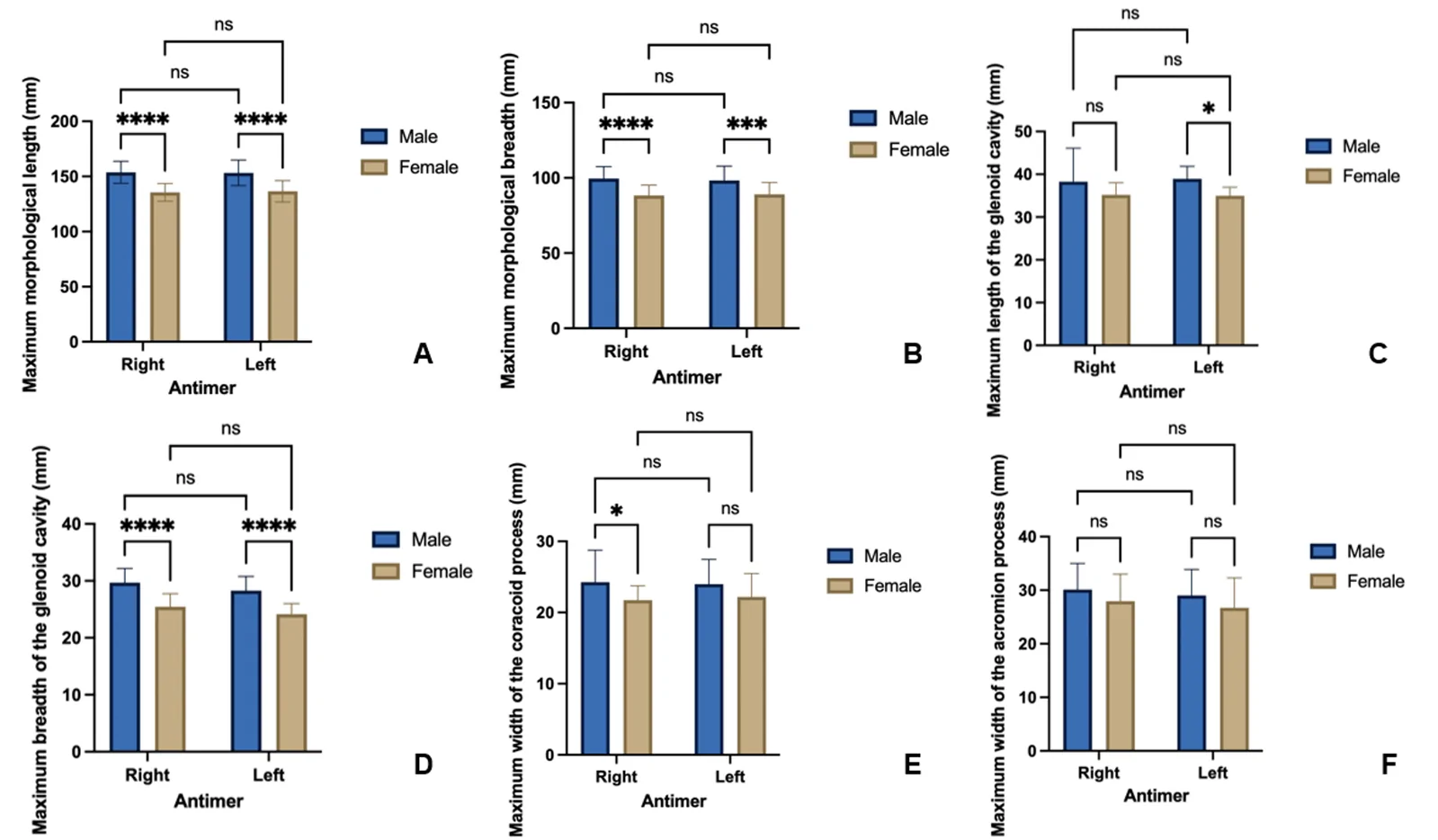
Multiple comparisons analysis of scapular measurements between sexes (male and female) and antimers. A) Maximum morphological length (MML); B) maximum morphological breadth (MMB); C) maximum length of the glenoid cavity (MLGC); D) maximum breadth of the glenoid cavity (MBGC); E) maximum width of the coracoid process (MWCP); and F) maximum width of the acromion process (MWAP). Statistical significance was determined using two-way ANOVA followed by Sidak’s multiple comparisons test. Data are presented as mean ± standard deviation. *p < 0.05; **p < 0.01; ***p < 0.001; ****p < 0.0001.

For the maximum morphological length (MML), statistically significant differences between sexes were observed on both the right (p<0.0001) and left antimers (p<0.0001), with no significant interaction between antimers within the same sex (Figure 2A).

Similarly, the maximum morphological breadth (MMB) showed statistically significant differences between males and females on both the right (p<0.0001) and left antimers (p=0.0007) (Figure 2B).

Regarding the maximum length of the glenoid cavity (MLGC), no statistically significant difference between sexes was observed for the right scapulae (p=0.613), whereas a significant difference was identified for the left scapulae (p=0.0131) (Figure 2C).

For the maximum breadth of the glenoid cavity (MBGC), statistically significant differences between sexes were found on both the right (p<0.0001) and left antimers (p<0.0001) (Figure 2D).

In the measurement of the maximum width of the coracoid process (MWCP), no statistically significant difference between sexes was observed for the left scapulae (p=0.1799); however, a significant difference was identified for the right scapulae (p=0.0387) (Figure 2E).

No statistically significant differences were observed for the maximum width of the acromion process (MWAP) regarding sex, antimer, or their interaction (Table 2, Figure 2F).

**Table 2:** Results of the two-way ANOVA evaluating the effects of sex, side, and their interaction on scapular morphometric measurements. The values in the Interaction, Side, and Sex columns are F-statistics from the analysis of variance (ANOVA).

Variable	Interaction F (1,84)	p-value	Side F(1,84)	p-value	Sex F(1,84)	p-value
MML	0.1002	0.7524	0.0106	0.9183	66.30	<0.0001
MMB	0.3162	0.5754	0.0171	0.8961	34.33	<0.0001
MLGC	0.1777	0.6745	0.0580	0.8102	12.40	0.0007
MBGC	0.0049	0.9442	7.276	0.0084	70.12	<0.0001
MWCP	0.2370	0.6277	0.0176	0.8947	8.291	0.0051
MWAP	0.0039	0.9506	1.099	0.2975	4.209	0.0433

## Discussion

Shoulder pain is a complaint that affects a proportionally large portion of the world's population [21]. Studies have highlighted that the morphology and morphometry of the scapula are directly related to dysfunctions in the shoulder joint and directly affect their treatment methods [5, 21-24]. Knowing the morphology and the morphometry of the scapula provides important information for clinical research and surgeries related to it, allowing for greater safety during surgeries and procedures involving this region [3].

The coracoid process is an important structure in the biomechanics of the shoulder joint, and its morphology and morphometry may influence the occurrence of subacromial impingement syndrome [23, 25]. Glenoid morphology and morphometry are relevant to the outcome of anatomical and reverse total shoulder arthroplasty, for shoulder prosthetic replacement surgery [5-6].

It is well known that human bones exhibit sexual dimorphism, which can be expressed through morphological and metric differences [8]. Specific measures for each population are necessary, as it is known that the accuracy of any method decreases when applied to a population other than the one for which it was initially developed [10]. The observed differences in skeletal morphology between populations have been attributed to several factors, including geography, climate, genetics, nutrition, and socioeconomic differences [1-2, 7-9, 19]. 

Thus, many studies have been conducted to verify whether the scapula presents sexual dimorphism in different populations, and it has been proven that the male scapula is generally larger than the female’s [1, 7-10, 12, 14-17, 19-20, 26]. The results obtained in the present study, in a sample of a population from southeastern Brazil, corroborate the findings of these previous studies, since male scapulae presented higher morphometric values compared to female scapulae. No statistically significant differences were observed between the right and left antimers within the same sex in any of the 6 measurements.

Considering the anatomy of the scapula, the most preserved bony features are the acromion process, the coracoid process, and the glenoid cavity [8, 19]. In the present study, no statistically significant differences were observed for the MWAP regarding sex, antimer, or their interaction. However, the MWCP showed a significant difference for the right scapulae (p=0.0387) when comparing sexes. Similarly, for the MLGC, a significant difference was identified for the left scapulae (p = 0.0131) when comparing sexes. For the MBGC, statistically significant differences between sexes were found on both the right (p<0.0001) and left antimers (p<0.0001). 

Mathews et al. [5] evaluated the glenoid cavity morphology in a sample of the Swiss population and showed that the maximum height of the glenoid cavity and the maximum width of the glenoid cavity had a significant sex dimorphism. The measurements in male scapulae were larger compared to the measurements in female scapulae [5].

Mangasah and Aminata [6] analyzed scapulae from an Indonesian population and showed that the maximum height of the glenoid cavity, the maximum width of the glenoid cavity, and the coracoid length had a significant sex dimorphism. For all measurements, the values ​​were higher in male scapulae compared to female scapulae [6].

Chaimongkhol et al. [25] studied the morphometry of the acromion in a Thai population and reported that there were statistically significant differences between sexes for all parameters, but no differences between right and left sides. Corroborating the other studies, males showed higher values than females for all parameters [25].

In the present study, for the MML, statistically significant differences between sexes were observed on both the right (p<0.0001) and left antimers (p<0.0001). Similarly, the MMB showed statistically significant differences between males and females on both the right (p<0.0001) and left antimers (p=0.0007). 

The difference in size and proportion observed when comparing male bones to female bones is a biological phenomenon that involves different factors, including evolutionary factors such as sexual selection and competition for resources, in addition to the influence of hormones, genetic characteristics, and environmental characteristics of different demographic groups [18, 27]. Furthermore, it should be considered that males have greater skeletal muscle mass attached to bones, resulting in a more robust male skeleton compared to the female skeleton [28].

The importance of conducting morphometric studies in different populations is highlighted, as anatomical variation may influence skeletal characteristics. Expanding the available data on different bones and measurement approaches contributes to a better understanding of morphological patterns when comparing the sexes. 

Limitations should be considered in this study. The sample size (n=88) is relatively small to assert direct application to the forensic context. Additionally, restricting the sample to a population from southeastern Brazil also limits the generalization of the results found, especially considering that this is a mixed-race population with heterogeneous ancestry and different socioeconomic contexts. All these factors can interfere with scapula morphology in relation to sexual dimorphism. Further studies, including larger and more diverse samples, are recommended to validate and expand these findings, particularly before a forensic application is considered.

## Conclusions

In conclusion, the results of the present study indicate that measurements of human scapulae from a sample of the southeastern Brazilian population demonstrate significant differences between the sexes, while no statistically significant differences were observed between the right and left antimers within the same sex. Due to anatomical variations across populations, further studies, including human scapulae from other regions, are recommended to verify whether these findings are consistent across different populations. 
